# Population and breast cancer patients’ analysis reveals the diversity of genomic variation of the BRCA genes in the Mexican population

**DOI:** 10.1186/s40246-018-0188-9

**Published:** 2019-01-10

**Authors:** J. C. Fernández-Lopez, S. Romero-Córdoba, R. Rebollar-Vega, L. A. Alfaro-Ruiz, S. Jiménez-Morales, F. Beltrán-Anaya, R. Arellano-Llamas, A. Cedro-Tanda, M. Rios-Romero, M. Ramirez-Florencio, V. Bautista-Piña, C. Dominguez-Reyes, F. Villegas-Carlos, A. Tenorio-Torres, A. Hidalgo-Miranda

**Affiliations:** 1 Laboratorio de Genómica del Cáncer, Instituto Nacional de Medicina Genómica, Perfiérico Sur, 4809, Arenal Tepepan, 14610 Mexico City, CP Mexico; 2 Instituto de Enfermedades la Mama FUCAM, Avenida El Bordo 100, Santa Ursula Coapa, 04980 Mexico City, CP Mexico

**Keywords:** BRCA, Breast cancer, Populations, Germline, Genetic testing

## Abstract

Interpretation of variants of unknown significance (VUS) in genetic tests is complicated in ethnically diverse populations, given the lack of information regarding the common spectrum of genetic variation in clinically relevant genes. Public availability of data obtained from high-throughput genotyping and/or exome massive parallel sequencing (MPS)-based projects from several thousands of outbred samples might become useful tools to evaluate the pathogenicity of a VUS, based on its frequency in different populations. In the case of the Mexican and other Latino populations, several thousands of samples have been genotyped or sequenced during the last few years as part of different efforts to identify common variants associated to common diseases. In this report, we analyzed Mexican population data from a sample of 3985 outbred individuals, and additional 66 hereditary breast cancer patients were analyzed in order to better define the spectrum of common genomic variation of the *BRCA1* and *BRCA2* genes. Our analyses identified the most common genetic variants in these clinically relevant genes as well as the presence and frequency of specific pathogenic mutations present in the Mexican population. Analysis of the 3985 population samples by MPS identified three pathogenic mutations in *BRCA1*, only one population sample showed a *BRCA1* exon 16–17 deletion by MLPA. This resulted in a basal prevalence of deleterious mutations of 0.10% (1:996) for *BRCA1* and 11 pathogenic mutations in *BRCA2*, resulting in a basal prevalence of deleterious mutations of 0.276% (1:362) for *BRCA2*, combined of 0.376% (1:265). Separate analysis of the breast cancer patients identified the presence of pathogenic mutations in 18% (12 pathogenic mutations in 66 patients) of the samples by MPS and 13 additional alterations by MLPA. These results will support a better interpretation of clinical studies focused on the detection of BRCA mutations in Mexican and Latino populations and will help to define the general prevalence of deleterious mutations within these populations.

**Electronic supplementary material:**

The online version of this article (10.1186/s40246-018-0188-9) contains supplementary material, which is available to authorized users.

## Introduction

*BRCA1* and *BRCA2* might represent two of the most characterized genes in the human genome due to their association with hereditary cancer syndromes. However, the complete spectrum of *BRCA* genetic variation among ethnically diverse populations has not been fully described. Germline mutations in these genes are mainly associated to familial breast and ovarian cancer and more recently with pancreatic and prostate cancer. In addition to the identification of subjects with a higher risk of developing cancer in a familial setting, detection of deleterious germline *BRCA* mutations is also required for the selection of treatment with PARP inhibitors, which can induce synthetic lethality in cancer cells in the presence of deleterious *BRCA* mutations. Currently, treatment with PARP inhibitors are approved for patients with deleterious or suspected deleterious germline *BRCA* mutations with *HER2*-negative metastatic breast cancer, for patients with hormone receptor-positive breast tumors that have been treated or are not susceptible to receive endocrine therapy [1], and in patients with advanced ovarian cancer who have been treated with three or more prior lines of chemotherapy [2].

The prevalence of combined mutations in the totality of the coding region of these genes vary between different countries and ethnic groups, being approximately 0.3% in Caucasian women in the USA and 2.5% in Jewish women living in Israel or in the USA. A recent paper focused on the analysis of exome sequencing-based screening for BRCA1/2 among adult biobank participants identified a higher frequency of pathogenic/likely pathogenic mutations than previous reports, identifying a 1:180 prevalence of deleterious mutations and suggesting that compared with previous clinical care, exome sequencing-based screening identified five times as many individuals with pathogenic or likely pathogenic *BRCA1/2* variants [3].

The prevalence of mutations in Latin American populations has not yet been fully defined. In Mexico, a limited number of analyses in the BRCA1 and BRCA2 genes have been carried out using different methods [4–10]. These studies have identified the presence of pathogenic germline mutations in 28% of the patients with ovarian cancer and 15% of the patients with breast cancer, without any selection for family history. The percentage of BRCA1 mutations in women diagnosed with triple negative breast tumors, without any selection for familial cancer, increases up to 28%. In patients with ovarian cancer, without any selection for familial cancer, the BRCA1 ex9-12del mutation was detected in 33% of the cases, supporting the notion that this is a founder mutation in Mexico [8, 10].

A recent review about mutations in the BRCA genes identified a clear founder effect in several Latin American populations, including Mexico (BRCA1 ex9–12del), Brazil (*BRCA1* 5382insC y *BRCA2* c.156_157insAlu), and Colombia (*BRCA1* 3450del4, A1708E, y *BRCA2* 3034del4), as well as in Latino population from southern California (*BRCA1* 185delAG, IVS5+1G>A, S955x, y R1443x). The differences in the frequency and type of *BRCA* mutations in Latin America have been associated with the admixture dynamics in each specific population and with the differences in the proportions of ancestral components resulting from the admixture processes over time [11].

However, the analysis of a much higher number of samples is necessary in order to define the frequency of pathogenic mutations and to define the whole spectrum of common genetic variation in clinically relevant genes in the Mexican population [12]. With the advent of massive parallel sequencing and the reduction of costs for sequencing-based diagnostic panels, this situation is already improving. Nonetheless, it will take time to have enough clinical samples to define the population-wide spectrum of common variation and to define a better threshold to evaluate pathogenicity based on allele frequency, as recommended by the American Colleague of Medical Genetics and Genomics and the Association for Molecular Pathology. To define this threshold, it is necessary to have population-based genotyping information, in order to determine if the allele frequency of a particular variant is “higher” than expected for the disorder, a situation that provides strong evidence to consider the variant benign [13]. Fortunately, thanks to the public availability of data obtained from high-throughput genotyping and/or massive parallel exome sequencing projects from several thousands of outbred samples (ExAC [14], 1000 genomes [15]), we can analyze the presence of specific variants in different populations in order to compare how common they are and to evaluate their potential pathogenicity depending on their allele frequency.

In the case of the Mexican and other Latino populations, several thousands of samples have been genotyped or sequenced during the last few years as part of different efforts to identify common variants associated to common diseases, such as diabetes (The Slim Initiative in Genomic Medicine for the Americas (SIGMA) T2D Consortium) [16–18]. This information has led to the identification of diabetes-related variants enriched in the Mexican population, but has also provided population-based frequencies of common genetic variants throughout the genome, information which can be used to define the spectrum of common genetic variation in clinically relevant genes.

In this report, we analyzed Mexican population data from a sample of 3985 outbred individuals, and additional 66 hereditary breast cancer patients were analyzed in order to better define the spectrum of common genomic variation of the *BRCA1* and *BRCA2* genes. Our analyses identified the most common genetic variants in these clinically relevant genes as well as the presence and frequency of specific pathogenic mutations present in the Mexican outbred population and corroborated the presence and frequency of pathogenic mutations in hereditary breast cancer patients. These results will support a better interpretation of clinical studies focused on the detection of BRCA mutations in Mexicans and Latino populations and will help to define the general prevalence of deleterious mutations within these populations.

## Materials and methods

### Studied population

All the procedures and protocols were reviewed and approved by the Ethics and Research Committee of the National Institute of Genomic Medicine (INMEGEN) and were compliant with the Helsinki declaration. *BRCA1* and *BRCA2* genetic variants data included in this study were obtained from 3985 outbred individuals from two sources: (1) the SIGMA Type 2 Diabetes Whole Exome Sequencing Project database and (2) open population samples from the Mexican Genome Diversity Project (MGDP), where the *BRCA1* and *BRCA2* genes were analyzed by massive parallel sequencing and multiplex ligation-dependent probe amplification (MLPA).

Additionally, 66 samples from women with breast cancer with history of familial cancer were also analyzed.

### Population samples, source 1: SIGMA Type 2 Diabetes Whole Exome Sequencing Project

There are 3842 unrelated individuals from the Slim Initiative in Genomics Medicine for the Americas Type 2 Diabetes Whole-Exome Sequence Project (SIGMA Type 2 Diabetes). Data is deposited in the type 2 diabetes knowledge portal [18].

### Population samples, source 2: Mexican Genome Diversity Project

There are 143 unrelated anonymous women with no associated phenotype, which were collected as part of the Mexican Genome Diversity Project (MGDP), as described by Silva-Solezzi, et al [19]. These samples were selected from the Mexican States of Campeche, Zacatecas, Sonora, Yucatán, Tamaulipas, Guerrero, Guanajuato, and Veracruz and are considered the “Mestizo” population (admixed). We also included samples from the Amerindian Tepehuano group, from Durango, the Zapoteco group from Oaxaca, and Mayas from Campeche.

### Women with breast cancer and history of familial cancer

Sixty-six samples from breast cancer patients with familial cancer history, which were identified by a clinical geneticist, were included in the study after informed consent at the Instituto de Enfermedades de la Mama FUCAM, AC.

Both the MGDP and the breast cancer patients were analyzed for *BRCA*1 and *BRCA*2 mutations by massive parallel sequencing at the National Institute of Genomic Medicine in Mexico City. Variants and frequencies of the *BRCA1* and *BRCA2* genes from individuals from the SIGMA project were identified as described in references [16, 17].

### Ancestral components of the SIGMA and MGDP samples

**Fig. 1:**
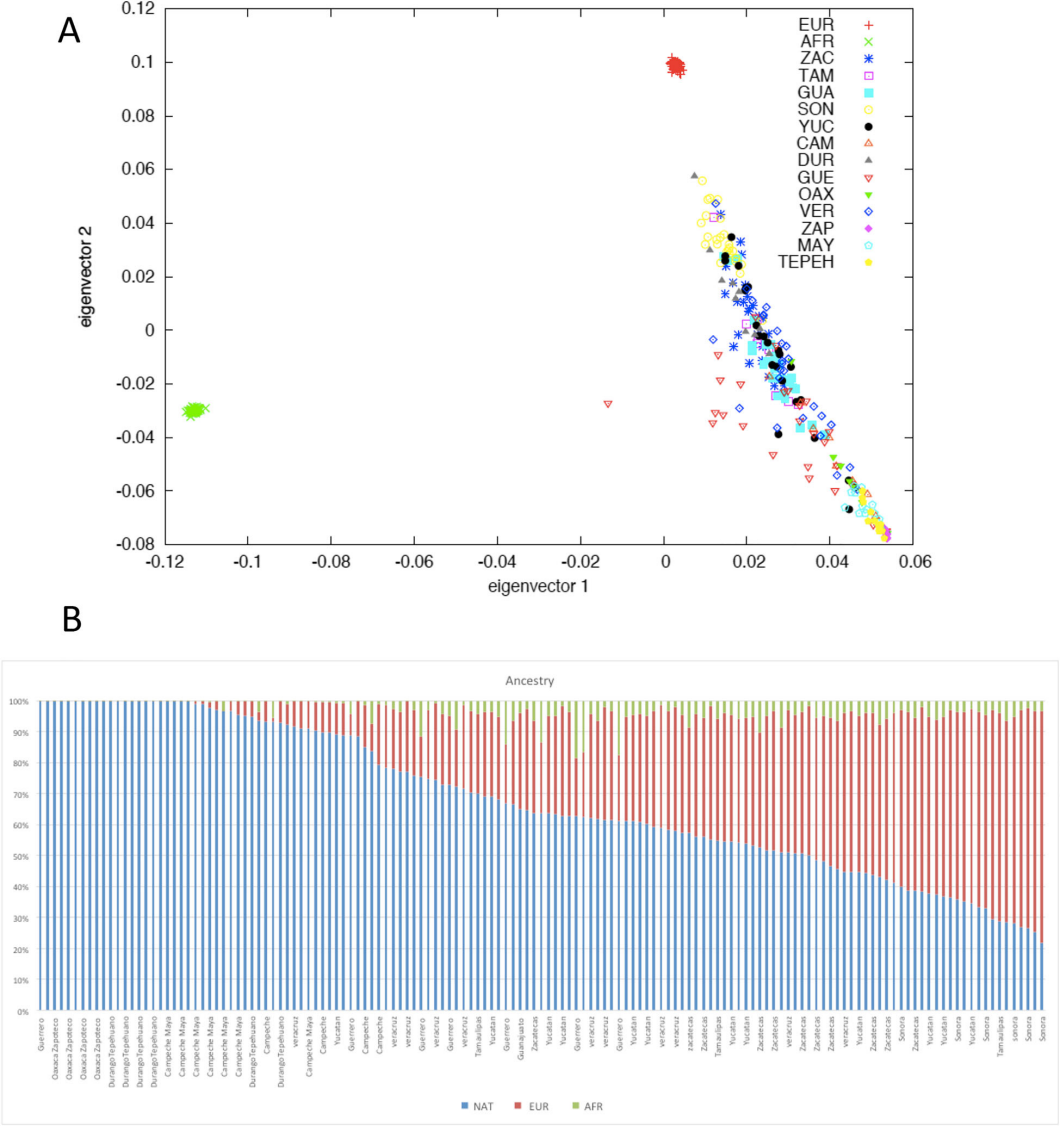
Principal component analysis of the Mexican Genome Diversity Project samples included. European and African samples from the International HapMap Project were included as references. **a** The PCA shows that the mestizo (admixed populations) are located between the Amerindian samples (ZAP, Zapoteco; MAY, Maya; TEPEH, Tepehuano) and the European samples, in a wide distribution defined by the percentage of Amerindian-European Ancestry. **b** Ancestry proportions of each sample

All data sets had either Mexican or other Latino ancestry based on self-reporting. This was corroborated using principal component analysis of genotype data. The average ancestry proportion of the Native American component in the SIGMA Type 2 Diabetes data set was 0.69. In the MGDP Mestizo samples, the ancestry components were Native American 0.59, European 0.37, and African 0.04. The Amerindian ancestry proportion in the Tepehuano, Zapoteco, and Maya samples was 0.97, 0.02 European, and 0.003 African. Figure 1 shows the principal components analysis (PCA) and ancestral component distribution of the Mexican Genome Diversity Project samples. Additional file 1: Table S1 lists the complete ancestral components of each of these samples.

### BRCA1 and BRCA2 massive parallel sequencing analysis

Genomic DNA from blood lymphocytes was purified with the QIAamp DNA Blood Maxi Kit. DNA was quantified and adjusted to a concentration of 5 ng/μL using a Qubit 2.0 Fluorometer (Invitrogen, Waltham, MA, USA) and the Qubit dsDNA HS Assay Kit (Invitrogen). For target enrichment, 50 ng of genomic DNA was amplified using custom primers designed to target all coding exons of *BRCA1* and *BRCA2*, and library preparation was done using the TruSeq HT library preparation kit (Illumina, San Diego, CA, USA) according to the manufacturer’s instructions. Pooled libraries were sequenced on a MiSeq sequencer (Illumina) using the MiSeq Reagent Kit v2 (300 cycles). Sequencing data was analyzed using Illumina’s BaseSpace TruSeq amplicon tools V3.0 and the variants that passed all quality controls were annotated using ClinVar and BRCAExchange.

### MLPA

Exonic deletions and duplications affecting the *BRCA* genes were detected on genomic DNA using the multiplex ligation-dependent probe amplification (MLPA) commercial kits from MRC-Holland, Amsterdam, The Netherlands, according to the manufacturer’s recommendations. The Coffalyser software (V.140721.1958) was used for data analysis.

### Annotation of variants

ClinVar [20] (28/02/18) and BRCAexhange (http://brcaexchange.org/ version 19/10/17) data were used to annotate all variants. Even though the BRCA exchange data includes allele frequency from ExAC, this version does not include the total number of Latino samples reported in the current version of ExAC, so we updated this information directly from the ExAC portal (http://exac.broadinstitute.org/downloads version 1.0 02/27/17). Only variants that passed the genotyping quality metrics in ExAC were included in the analysis.

### Availability of data and materials

The datasets supporting the conclusions of this article are included within the article and its additional files. These data include all the variants detected in the population samples analyzed in this study.

## Results

### Frequency of BRCA mutations in the Mexican outbred population samples

From a sample consisting of 3985 population samples (143 sequenced in this study and 3842 from the SIGMA study), we identified 15 pathogenic mutations (3 detected by massive parallel sequencing and 1 by MPLA in *BRCA1* and 11 in *BRCA2*, all detected by sequencing), resulting in a population frequency of deleterious mutations of 0.10% (1:996) for *BRCA1* and 0.276% (1:362) for *BRCA2*, combined of 0.376% (1:265).

Table 1 shows the 15 pathogenic mutations identified in the open population samples for *BRCA1* and *BRCA2*.

**Table 1:** Pathogenic mutations identified in the 3842 sample SIGMA dataset. Fifteen pathogenic mutations (4 in *BRCA1* and 11 in *BRCA2* were identified in our population-based analysis

SNP	Position	Ref	Alt	Allele frequency Mexicans	Annotation	HGVS_cDNA_LOVD	Allele frequency (EXAC)	Allele frequency African	Allele frequency East Asian	Allele frequency European (Non-Finish)	Allele frequency Finnish	Allele frequency Latino	Allele frequency Other	Allele frequency South Asian	Clinical significance
*BRCA1*															
Rs28897696	17:41215920	G	T	0.0001301	Missense	NM_007294.3:c.5123C>A	0.00002487	0	0	0.0000302	0	0.0000869	0	0	Pathogenic
Rs41293455	17:41234451	G	A	0.0001301	Stop gained	NM_007294.3:c.4327C>T	0.00001648	0	0	0	0.000151194	0.0000864	0	0	Pathogenic
Rs80357902	17:41243899	A	AT	0.0001301	Frameshift	NM_007294.3:c.3648dupA	0.000008244	0	0	0	0	0.0000867	0	0	Pathogenic
MLPA	BRCA1 ex16–17del														
*BRCA2*															
rs80359775	13:32972346	TTGTA	T	0.0007808	Frameshift	c.9699_9702del	0.00009916	0	0	0.000045	0	0.000780302	0	0	Pathogenic
Rs80359335	13:32911080	AT	A	0.0001302	Frameshift	c.2589del	0.000008311	0	0	0	0	0.0000873	0	0	Pathogenic
rs11571658	13:32914766	CTT	C	0.0001301	Frameshift	c.6275_6276del	0.00001667	0	0	0.0000151	0	0.0000865	0	0	Pathogenic
Rs587782428	13:32954260	CG	C	0.0001301	Frameshift	c.9235del	0.000008269	0	0	0	0	0.0000867	0	0	Pathogenic
rs80358494	13:32910716	C	T	0.0001301	Stop gained	c.2224C>T	0.000008254	0	0	0	0	0.000087	0	0	Pathogenic
rs80359082	13:32944584	G	A	0.0001301	Missense	c.8377G>A	0.000008237	0	0	0	0	0.0000864	0	0	Pathogenic
rs80359418	13:32890599	T	G	0.0001301	Start lost	c.2 T>G	0.000008339	0	0	0	0	0.0000867	0	0	Pathogenic
rs80359519	13:32914033	CA	C	0.0001301	Frameshift	c.5542del	0.000008279	0	0	0	0	0.0000866	0	0	Pathogenic
rs80359604	13:32903604	TGT	T	0.0001301	Frameshift	c.658_659del	0.00006119	0.000119076	0	0.0000783	0	0.000100908	0	0	Pathogenic
Rs876660636	13:32914122	AC	A	0.0001301	Frameshift	c.5631del	0.000008296	0	0	0	0	0.0000867	0	0	Pathogenic
Rs878853620	13:32972336	CT	C	0.0001301	Frameshift	c.9689del	0.000008283	0	0	0	0	0.0000868	0	0	Pathogenic

### Identification of BRCA variants in the Mexican population

We identified 160 variants in *BRCA1* and 274 variants in *BRCA2* based on the analysis of 3842 Mexican population samples from the SIGMA Diabetes database.

Regarding the 160 variants in *BRCA1*, 52 were benign, 33 were benign-likely benign, 23 had conflicting interpretations of pathogenicity, 18 were of uncertain significance, and only 3 were pathogenic (NM_007294.3:c.5123C>A, NM_007294.3:c.4327C>T, NM_007294.3:c.3648dupA). Twenty-nine variants are not found in ClinVar and have not been yet reviewed by the ENIGMA consortium, out of these, one affects a splice region and eight are missense mutations. One mutation (NM_007294.3:c.1729G>C) is predicted as possibly damaging by polyphen (score 0.77). Of the 160 variants, 69 have only been reported in the Latino population from ExAC and might represent Latino-specific variants.

Regarding *BRCA2*, 274 variants were found, of which, 143 were missense, 51 synonymous, two generated a stop gain, one a start loss, five were in splice regions, one in non-coding transcript exons, 54 were in introns, one was an inframe insertion, one an inframe deletion, eight frameshift and four were in the 3′ and 5′ regions.

Eleven of the mutations found in *BRCA2* in the population dataset were classified as pathogenic by the ENIGMA consortium or ClinVar. Thirty-seven variants were not in ClinVar and have not been reviewed by ENIGMA. Eight of these were missense and three (NM_000059.3 c.2635T>C; NM_000059.3 c.6416A>T; NM_000059.3 c.8816A>G) were predicted as probably damaging by polyphen (score 0.996).

From a population standpoint, 121 of the 174 variants detected in the Mexican subjects from SIGMA project have not been observed in other groups and are also private of the Latino population in ExAC. These seemingly Latino private variants include six of the ten pathogenic mutations in *BRCA2,* a situation that is confirmed by the ethnicity report of the ClinVar submitters reporting some of these mutations. They are all low-frequency mutations which are present as heterozygotes in one individual out of the approx. 3842 samples analyzed, except for one (rs80359775) which is present as heterozygote in six individuals.

Regarding variants of unknown significance in *BRCA2*, 106 of the 274 variants were classified as either “uncertain significance” or “conflicting interpretations of pathogenicity.” Nineteen were missense mutations classified as possibly or probably deleterious by Polyphen or SIFT and showed a low-allele frequency on all populations suggesting they might probably represent pathogenic variants.

Additional file 2: Table S2 and Additional file 3: Table S3 show the complete list of variants detected in all samples, together with their annotation.

### Open population from Mexican genome diversity Project

The 143 open population samples from the MGDP did not present any pathogenic mutation, except for one sample with a *BRCA1* x16–17del deletion (Additional file 4: Table S4). Variants with the highest allele frequency identified in the SIGMA database were also between the most common identified in the MGDP sample. Additional file 4: Table S4 shows the MLPA results.

### Mutation analysis in breast cancer patients

Sixty-six samples with a suspected history of familial cancer were sequenced. Massive parallel sequencing identified pathogenic mutations in 12 samples (18%, Table 2), two of these were also found in the open population samples (NM_007294.3:c.4327C>T; NM_007294.3:c.3648dupA). Seven additional mutations classified as uncertain significance were classified as “probably damaging” by polyphen.

**Table 2:** Pathogenic mutations identified in the breast cancer patients

Sample ID	RS	GENE	POS	REF	ALT	Clinical_significance_ENIGMA	HGVS_cDNA	REFERENCE
FUCAM29	rs80357520	BRCA1	41243787	TTA	T	Pathogenic	c.3759_3760delTA	Villareal-Garza 2015 [8]
FUCAM50	rs41293455	BRCA1	41234451	G	A	Pathogenic	c.4327C>T	McKean-Cowdin 2005 [24]
FUCAM53	rs80357382	BRCA1	41258474	T	C	Pathogenic	c.211A>G	Rebbek 2016 [25]
FUCAM56	rs80357780	BRCA1	41245250	ACT	A	Pathogenic	c.2296_2297delAG	Weitzel 2005 [26]
FUCAM65	rs80357902	BRCA1	41243899	A	AT	Pathogenic	c.3648dupA	Lecarpentier 2012 [27]
FUCAM75	rs80357842 rs80357889	BRCA1	41243686	CCTCA	C	Pathogenic	c.3858_3861delTGAG	Kwong 2016 [28]
FUCAM77	rs80357914	BRCA1	41276044	A	ACT	Pathogenic	c.68_69delAG	Bolton 2012 [29]
FUCAM1	rs777107618 rs80359380	BRCA2	32911755	C	CT	Pathogenic	c.3264dupT	Susswein 2016 [29]
FUCAM36	rs41293513	BRCA2	32937507	A	G	Pathogenic	c.8168A>G	Guidugli 2013 [30]
FUCAM40	rs80359660	BRCA2	32930683	G	GC	Pathogenic	c.7556dupC	Borg 2010 [31]
FUCAM48	rs80359082	BRCA2	32944584	G	A	Pathogenic	c.8377G>A	Guidugli 2013 [30]
FUCAM310	rs80359082	BRCA2	32944584	G	A	Pathogenic	c.8377G>A	Guidugli 2013 [30]
MLPA								
FUCAM33		BRCA1					Ex9-12del	
FUCAM11		BRCA1					Ex9-11del	
FUCAM30		BRCA1					Ex12del	
FUCAM84		BRCA2					Ex1del	
FUCAM85		BRCA2					Ex23del	
FUCAM98		BRCA2					Ex1del	
FUCAM100		BRCA2					Ex17del	
FUCAM102		BRCA2					Ex23del	
FUCAM9		BRCA2					Ex22-24del	
FUCAM10		BRCA2					Ex22-24del	
FUCAM25		BRCA2					Ex11del	
FUCAM41		BRCA2					Ex26del	
FUCAM47		BRCA2					Ex1del	

### MLPA

As described before, 49 population samples were analyzed for *BRCA1*, only one population sample showed a deletion in exons 16–17 (BRCA1 ex16–17del). For *BRCA2*, MLPA analysis did not find alterations in any of the 55 open population samples analyzed.

In 55 patients with history of familial breast cancer, the founder BRCA1 ex9–12del was identified in two samples and a third sample with familial history showed a BRCA1 ex12deletion.

In the 64 samples with history of familial breast cancer, BRCA2 exon 1 deletions were found in three cases, deletions of exon 11 in one case, deletion of exon 23 in two cases, exon 17 and exon 26 were deleted in one case each, and two samples presented BRCA2 ex22–24 deletions. MLPA results from these cases are presented in Additional file 4: Table S4.

Pathogenic mutations found in our dataset were identified as such in the BRCA Exchange database, based on the clinical significance defined by the ENIGMA consortium. The population frequencies were obtained from our data from the Mexican population and from other populations from the ExAC database. In both cases, the observed frequency of this allele is very low. However, the pathogenicity of these mutations is supported in several ways, including their report by several submitters, a good segregation with disease, deleterious effects on protein structure, analyzed in silico, and a high posterior probability of pathogenicity from multifactorial likelihood analysis.

## Discussion

Precise results interpretation of genomic testing is of paramount importance, both for the clinical management of the patients and to avoid unnecessary stress derived from an uncertain result. Erroneous interpretation of genetic data, such as when a patient is incorrectly informed that one of his or her variants is causal when in fact it is benign, have important adverse consequences for the patients and for their families.

A better interpretation of these analyses requires the inclusion of populations of diverse ethnical backgrounds, both through access to the tests themselves and also as part of scientific efforts aimed to describe human genomic diversity and its role in human disease. Several examples clearly show that this lack of representation already represents an important clinical problem for the interpretation of genetic tests [21], resulting in what has been called a “double disparity” where access to testing is limited and the interpretation of results are complicated by the lack of data from populations with a non-European background [22].

In Mexico, access to genetic testing for hereditary breast and ovarian cancer is still not widely available, limiting the amount of patient-derived data necessary to refine interpretation of their results. Fortunately, data generated from the research front is helping to mitigate this problem. In the last years, high-throughput genotyping and whole exome/genome sequencing efforts have included outbred samples from diverse ethnical populations, offering the possibility to contrast the frequency of suspicious variants against open population frequencies. These efforts include the Mexican genome diversity project [19, 23] and, more recently, exome sequencing projects aimed to the identification of common variants associated to diabetes and other diseases [16–18]. Recent studies have shown that this exome-based population approach might be more efficient in the identification of the frequency of pathogenic or likely pathogenic *BRCA1/2* mutations, being able to identify five times as many individuals with deleterious mutations compared to studies focused on selected populations in the clinical care [3].

Based on this data, in this paper, we describe the spectrum of common genomic variation in the *BRCA* genes in the Mexican population. Our analyses allowed us to identify variants that are enriched in the Mexican and Latino populations and to identify the identity and frequency of pathogenic mutations present in open population samples.

From a sample consisting of 3985 population samples (143 sequenced in this study and 3842 from the SIGMA study), we identified 15 pathogenic mutations (3 detected by massive parallel sequencing and 1 by MPLA in BRCA1 and 11 in BRCA2, all detected by sequencing), resulting in a population frequency of deleterious mutations of 0.10% (1:996) for BRCA1 and 0.276% (1:362) for BRCA2, combined of 0.376% (1:265). This is similar to what has been reported for population frequency carriers in ExAC without The Cancer Genome Atlas (TCGA) samples: 0.15% (1:646) for *BRCA1* and 0.26% for *BRCA2* (1:390; combined 0.41% 1:243). In 2016, the total population in Mexico was of 127.5 million, escalating the frequency of mutations, resulting in approximately 481,132 carriers of deleterious *BRCA* mutations among the Mexican population.

In order to evaluate the potential pathogenicity of variants with uncertain clinical significance, based on population-based data, we reviewed both the frequency and filtering allele frequency of the seven VUS detected in our breast cancer patients. The low frequency of an allele might be a criterion suggesting variant pathogenicity, but since frequency alone is not sufficient to define association with disease, we also reviewed their statistical threshold to filter them out if they are too common in the population to be associated with disease, based on the ExAC data (filtering AF in the ExAC browser). Four variants were filtered: rs80358861 (in European non-Finish), rs80359018 (Latino), rs80357323 (African), rs80358877 (Latino), but still, for two additional variants (rs80358947, rs80358621), this approach was not possible, since they are not described ExAC and have not yet been reviewed by ENIGMA). Sixty-seven additional variants were identified in the breast cancer patients which have not been described in BRCA Exchange and are not present in ExAC and might represent private polymorphisms.

Our sample collection reflects the genomic diversity of the Mexican population, based on the sample distribution in our PCA analysis, and we did not observed enrichment of particular variants regardless of the state of origin of the sample. We expected to see an enrichment of European-associated variants in the Northern states of Mexico (Sonora) or in samples with a higher percentage of European ancestral component. This suggests that the distribution of *BRCA* variants among the Mexican population might not differ significantly throughout different regions of the country. In fact, our dataset is particularly enriched in samples with a higher percentage of Amerindian ancestry (75% of Amerindian ancestral component on average); this would more likely represent variants which are enriched in in the Amerindian population, and this is supported by the observation that most of the variants detected in our population-based analysis were only present on the Latino population of ExAC.

In conclusion, our analyses allowed us to better define the common genomic variation of the *BRCA* genes in the Mexican open population, identifying specific pathogenic mutations and allowing the first calculation of the mutation burden in these clinically relevant genes in Mexico. Given the observed enrichment of these mutations the Latino population, our data will also be helpful to improve the interpretation of *BRCA1* and *BRCA2* mutation tests in other Latin American countries.

## Additional files

Additional file 1: **Table S1.** Ancestry information of all Mexican Genome Diversity Project samples included in the sequencing analysis. (XLSX 34 kb)

Additional file 2: **Table S2.** All variants detected in BRCA1 in the 3842 SIGMA samples, together with their annotation. (XLSX 254 kb)

Additional file 3: **Table S3.** All variants detected in BRCA2 in the 3842 SIGMA samples, together with their annotation. (XLSX 455 kb)

Additional file 4: **Table S4.** MLPA analysis of the Breast cancer samples. (XLSX 37 kb)
